# In this month’s Bulletin

**DOI:** 10.2471/BLT.25.000225

**Published:** 2025-02-01

In the editorial section, Viroj Tangcharoensathien et al. (82) introduce this special issue to accompany the 2025 Prince Mahidol Award Conference on digital technology to improve health services. Alain Labrique (83) explains why digital foundations are essential to the progression from infrastructure to impact in the health sector.

Gary Humphreys (86–87) reports the use of teleradiology supported by artificial intelligence in resource-constrained settings. Smisha Agarwal talks to Humphreys (88–89) about the challenges faced in introducing digital tools in such contexts.

## Afghanistan, Kenya, Pakistan, United Republic of Tanzania

### Getting an immediate second opinion

Asad Latif et al. (90–98) roll out peer-to-peer teleconsultations for critical care.

## Armenia, Bangladesh, Brazil, China, Colombia, Ethiopia, Ghana, India, Malawi, Malaysia, Nigeria, Pakistan, Philippines, Sri Lanka, Turkïye, Uganda

### Health workers’ adoption of digital health technology

Minmin Wang et al. (126–135) review the evidence for barriers and facilitators.

## Canada, China, Denmark, Egypt, France, Germany, Italy, Kingdom of the Netherlands, Spain, Thailand, United States of America

### Managing rheumatic diseases

Anindita Santosa et al. (136–147) analyse randomized controlled trials of digital interventions.

## China

### Reaching people living with HIV through primary health care 

Jun Wang et al. (99–109) implement digital services.

## India

### More efficient outpatient visits

Sutapa B Neogi et al. (164–169) describe a scan and share programme that reduced waiting times.

## Global

### What are digital determinants of health?

Robin van Kessel et al. (110–125) report findings from an evidence review and consensus process.

### Changing provision of services in low- and middle-income countries

Dominique J Monlezun et al. (148–154) discuss issues associated with health sector digitalization.

### Recessionary pressures and population health

Jo-An Occhipinti et al. (155–163) consider economic consequences of artificial intelligence.

### Designing a digital health ecosystem

Yao Xie et al. (170–173) list factors affecting inclusivity.

### Digital health diplomacy 

Jarbas Barbosa da Silva Jr et al. (174–176) make a case for universal health coverage.

